# Evaluation of Pulmonary Toxicity of Zinc Oxide Nanoparticles Following Inhalation and Intratracheal Instillation

**DOI:** 10.3390/ijms17081241

**Published:** 2016-08-01

**Authors:** Yasuo Morimoto, Hiroto Izumi, Yukiko Yoshiura, Taisuke Tomonaga, Takako Oyabu, Toshihiko Myojo, Kazuaki Kawai, Kazuhiro Yatera, Manabu Shimada, Masaru Kubo, Kazuhiro Yamamoto, Shinichi Kitajima, Etsushi Kuroda, Kenji Kawaguchi, Takeshi Sasaki

**Affiliations:** 1 Department of Occupational Pneumology, Institute of Industrial Ecological Sciences, University of Occupational and Environmental Health, 1-1 Iseigaoka, Yahata-nishi-ku, Kitakyushu, Fukuoka 807-8555, Japan; h-izumi@med.uoeh-u.ac.jp (H.I.); y-yoshiura@med.uoeh-u.ac.jp (Y.Y.); t-tomonaga@med.uoeh-u.ac.jp (T.T.); 2 Department of Environmental Health Engineering, Institute of Industrial Ecological Sciences, University of Occupational and Environmental Health, 1-1 Iseigaoka, Yahata-nishi-ku, Kitakyushu, Fukuoka 807-8555, Japan; toyabu@med.uoeh-u.ac.jp (T.O.); tmyojo@med.uoeh-u.ac.jp (T.M.); 3 Department of Environmental Oncology, Institute of Industrial Ecological Sciences, University of Occupational and Environmental Health, 1-1 Iseigaoka, Yahata-nishi-ku, Kitakyushu, Fukuoka 807-8555, Japan; kkawai@med.uoeh-u.ac.jp; 4 Department of Respiratory Medicine, School of Medicine, University of Occupational and Environmental Health, 1-1 Iseigaoka, Yahata-nishi-ku, Kitakyushu, Fukuoka 807-8555, Japan; yatera@med.uoeh-u.ac.jp; 5 Department of Chemical Engineering, Hiroshima University, Higashi-Hiroshima 739-8528, Japan; smd@hiroshima-u.ac.jp (M.S.); mkubo@hiroshima-u.ac.jp (M.K.); 6 National Institute of Advanced Industrial Science and Technology (AIST), 1-1-1 Higashi, Tsukuba, Ibaraki 305-8565, Japan; k-yamamoto@aist.go.jp (K.Y.); k-kawaguchi@aist.go.jp (K.K.); takeshi.sasaki@aist.go.jp (T.S.); 7 National Sanatorium Hoshizuka Keiaien, 4204 Hoshizuka-cho, Kanoya, Kagoshima 893-8502, Japan; skita-kufm@umin.ac.jp; 8 Laboratory of Vaccine Science, WPI Immunology Frontier Research Center, 6F IFReC Research Building, Osaka University, 3-1 Yamada-oka, Suita, Osaka 565-0871, Japan; kuroetu@ifrec.osaka-u.ac.jp

**Keywords:** zinc oxide, nanoparticle, CINC, neutrophil, intratracheal instillation, lung, inhalation

## Abstract

We conducted inhalation and intratracheal instillation studies of zinc oxide (ZnO) nanoparticles in order to examine their pulmonary toxicity. F344 rats were received intratracheal instillation at 0.2 or 1 mg of ZnO nanoparticles with a primary diameter of 35 nm that were well-dispersed in distilled water. Cell analysis and chemokines in bronchoalveolar lavage fluid (BALF) were analyzed at three days, one week, one month, three months, and six months after the instillation. As the inhalation study, rats were exposed to a concentration of inhaled ZnO nanoparticles (2 and 10 mg/m^3^) for four weeks (6 h/day, 5 days/week). The same endpoints as in the intratracheal instillation study were analyzed at three days, one month, and three months after the end of the exposure. In the intratracheal instillation study, both the 0.2 and the 1.0 mg ZnO groups had a transient increase in the total cell and neutrophil count in the BALF and in the expression of cytokine-induced neutrophil chemoattractant (CINC)-1, CINC-2, chemokine for neutrophil, and heme oxygenase-1 (HO-1), an oxidative stress marker, in the BALF. In the inhalation study, transient increases in total cell and neutrophil count, CINC-1,-2 and HO-1 in the BALF were observed in the high concentration groups. Neither of the studies of ZnO nanoparticles showed persistent inflammation in the rat lung, suggesting that well-dispersed ZnO nanoparticles have low toxicity.

## 1. Introduction

Various applications of nanomaterials, including metal oxide nanoparticles, have been enabled by new characteristics that have resulted from the progress of nanotechnology. Zinc oxide (ZnO) nanoparticles are white powders that are widely used in cosmetics, paint pigment, rubber additives, pharmaceutical products, and electronic materials. Many in vitro studies [1,2,3] and in vivo studies [4,5,6] have concluded that ZnO nanoparticles have a strong potential of toxicity, but these results are insufficient and controversial because the endpoints of toxicity in many such studies reflect acute responses, such as cytotoxicity and acute inflammation.

In lung disorders caused by inhaled particle, phagocytosis of inhaled particle induces infiltration of neutrophils and alveolar macrophages, and sustained or progressive inflammation is likely to cause lung injury and lead to irreversible chronic lesions, such as fibrosis and tumors [7,8,9]. Persistent inflammation, reported in animal exposure models using asbestos and silica, is important in the pathology of the formation of irreversible chronic lesions [7,9]. Most of the reports on ZnO nanoparticles show acute pulmonary inflammation in vivo and cytotoxicity in vitro, suggesting that ZnO nanoparticles may have harmful effects on humans [1,2,3,4,6,7]. However, it is also important to examine chronic responses, such as persistent inflammation. There are reports [10,11] that exposure to crystalline silica, a material known to have high toxicity, induced the onset of pulmonary inflammation after a certain observation time and more severe inflammation in the chronic phase. Considering the pulmonary toxicity of nanomaterials, it is important to evaluate the endpoints, such as inflammation and fibrosis, not only in the acute but also in the chronic phase. Therefore, we performed intratracheal instillation and inhalation studies of ZnO nanoparticles with more than three months of observation periods and examined pulmonary inflammation and fibrosis as the endpoints of toxicity in order to examine the pulmonary toxicity of ZnO nanoparticles.

## 2. Results

### 2.1. Intratracheal Instillation Study

#### 2.1.1. Cell Analysis in Bronchoalveolar Lavage Fluid (BALF)

**Figure 1:**
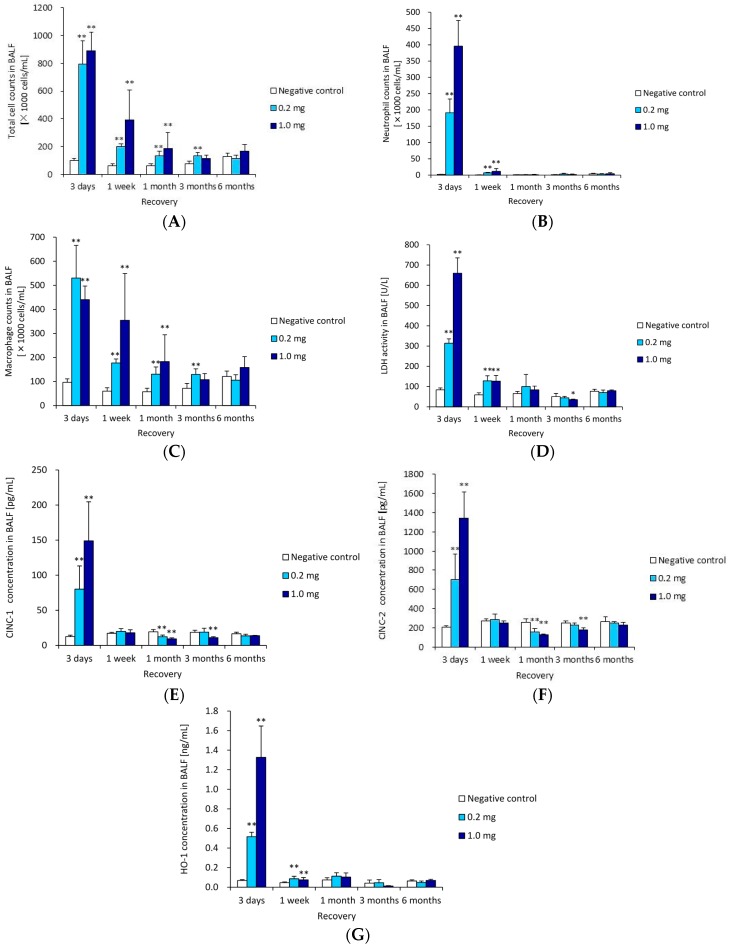
Cell number and cytokine level in bronchoalveolar lavage fluid (BALF) following intratracheal instillation of ZnO nanoparticles. (**A**) Total cell count in BALF; (**B**) neutrophil count in BALF; (**C**) macrophage count of in BALF; (**D**) lactate dehydrogenase (LDH) activity in BALF; (**E**) concentration of chemoattractant (CINC)-1 in BALF; (**F**) concentration of CINC-2 in BALF; and (**G**) concentration of heme oxigenase-1 (HO-1) in BALF. Intratracheal instillation of ZnO nanoparticles induced transient influx of inflammatory cells and expression of CINC-1, CINC-2 and HO-1 in BALF. * indicates *p* < 0.05 compared to negative control. ** indicates *p* < 0.01 compared to negative control.

Figure 1 shows the cellular analysis of the BALF following the intratracheal instillation of ZnO nanoparticles. The total cell count and neutrophil counts in the BALF were significantly and dose-dependently higher in the 1 mg group from three days post exposure compared with the negative control. The peak level of these counts was at three days, and they returned to nearly the level of the negative control according to a time course. The macrophage counts in the BALF were also higher in the ZnO groups than in the negative control at three days post exposure, although not dose-dependently. This response was also transient. A transient increase in the released Lactate Dehydrogenase (LDH) activity was observed in the 0.2 and 1 mg groups. This LDH activity was high, but decreased thereafter to nearly the same level as the negative control groups after one month.

#### 2.1.2. Cytokine-Induced Neutrophil Chemoattractant (CINC) Concentration in BALF

Figure 1E,F show the concentrations of CINC-1 and CINC-2 in the BALF following the intratracheal instillation of ZnO nanoparticles. The concentrations of CINC-1 were dose-dependently high in both the 0.2 and the 1 mg groups at three days post exposure, but at one month and three months post exposure, the value of CINC-1 in the ZnO groups was lower than that in negative control group. The concentrations of CINC-2 in the 0.2 and 1 mg groups were transiently higher than in the negative control group at three days post exposure, as like the concentration of CINC-1, and at one month and three months post exposure, the value of CINC-2 in the ZnO groups was lower than that in the negative control group.

#### 2.1.3. Heme Oxigenase-1 (HO-1) Concentration in BALF

Figure 1G shows the concentration of HO-1 in the BALF following the intratracheal instillation of ZnO nanoparticles. The concentration increased at three days post exposure, but there was no difference compared to the negative control group during the observation period after three days.

#### 2.1.4. Histopathological Changes in the Lungs

**Figure 2:**
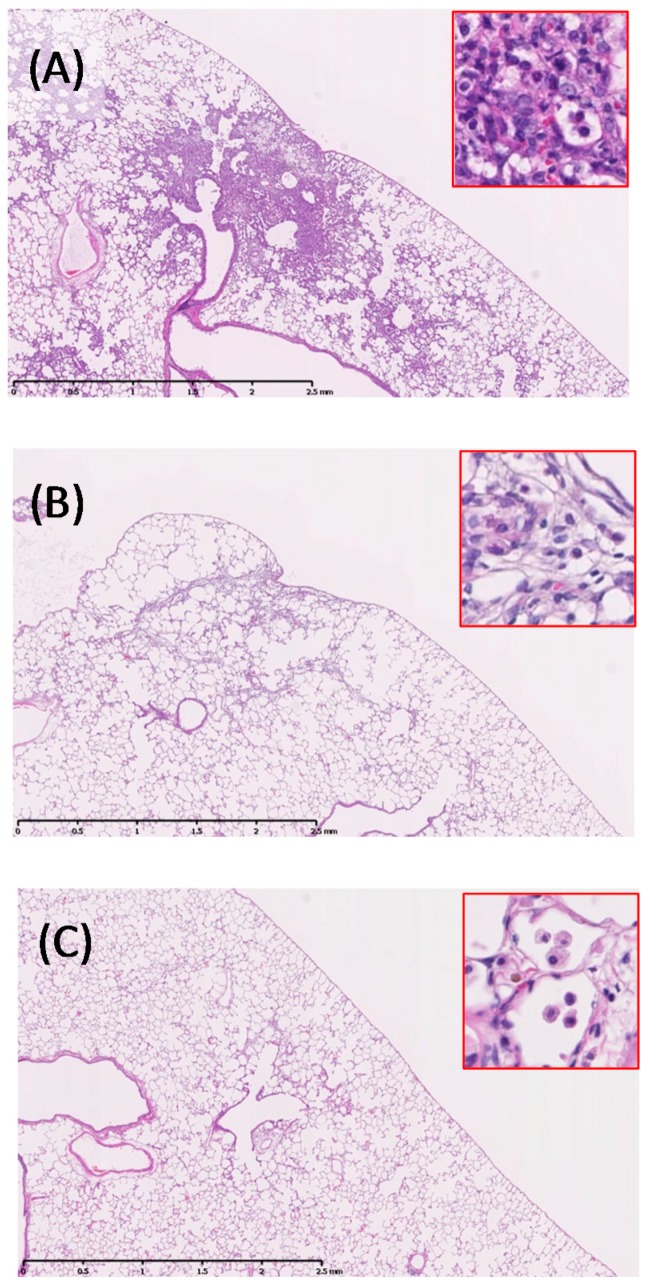
Histological changes in lungs of 1.0 mg-administered group (40×, inset 200×). (**A**) three days post exposure; (**B**) one month post exposure; and (**C**) three months post exposure. Bronchopneumonia was observed three days after intratracheal instillation of ZnO nanoparticles.

The lung specimens on day three showed infiltration of macrophages and inflammatory cells in the alveoli around the terminal bronchioles (Table 1). More inflammatory cell infiltration was observed in the lungs of the ZnO 1 mg instillation rats (Figure 2A) than in those of the 0.2 mg installation rats. Particle-laden macrophages were observed among the inflammatory cells, but the inflammation diminished one month after installation (Figure 2B). Minimal fibrosis occurred after inflammation, which disappeared three months after instillation. Some particle-laden macrophages were distributed around the alveolar ducts and the surrounding alveolar spaces.

#### 2.1.5. Morphological Features of Alveolar Macrophages by TEM

**Figure 3:**
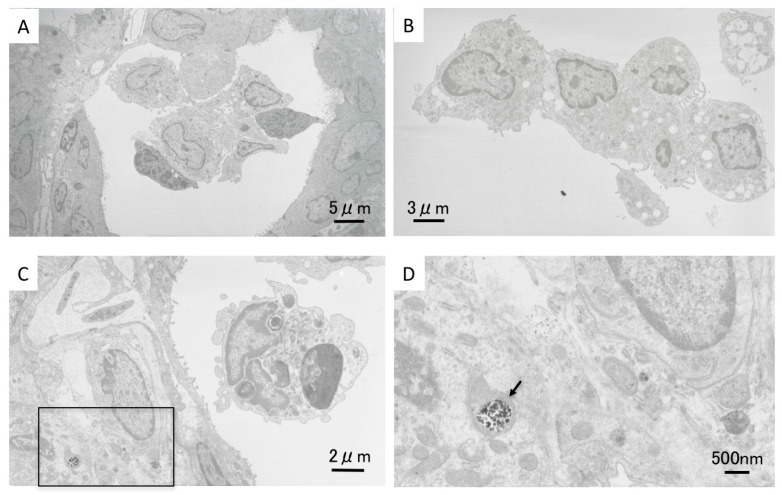
Lung tissue TEM images in the high dose exposed group at three days following intratracheal instillation. (**A**) Accumulation of alveolar macrophages and neutrophil cells in alveolar space; (**B**) accumulation of alveolar macrophages with vacuoles; (**C**) neutrophil cell and inflammatory cells; and (**D**) magnified image of boxed area in (**C**). Arrow: black particles formed aggregates in the cell organelles.

Figure 3A–D shows TEM images of the alveolar space near the inflammation in the high dose ZnO instillation group lung tissue at three days after exposure. Accumulation of alveolar macrophages and neutrophil cells can be seen (Figure 3A). TEM images of the inflammation are shown in Figure 3C, and neutrophil cells can be observed in the alveolar space. Figure 3D is a magnified image of the boxed area in Figure 3C. Black particles formed aggregates in the cell organelles, as indicated by the arrow. The shape of these nanoparticles indicate that they are ZnO nanoparticles, and that instilled ZnO nanoparticles reached the alveolar space. Figure 3B also shows an accumulation of alveolar macrophages, and many vacuoles can be observed. In our previous studies on the intratracheal instillation of NiO, TiO_2_, and CeO_2_ nanoparticles into rat lung, uptake of nanoparticles into alveolar macrophages was observed, but no ZnO nanoparticles were observed there in the present study. We speculate that ZnO nanoparticles were dissolved in the alveolar macrophages.

## 3. Inhalation Study

### 3.1. Cell Analysis in BALF

**Figure 4:**
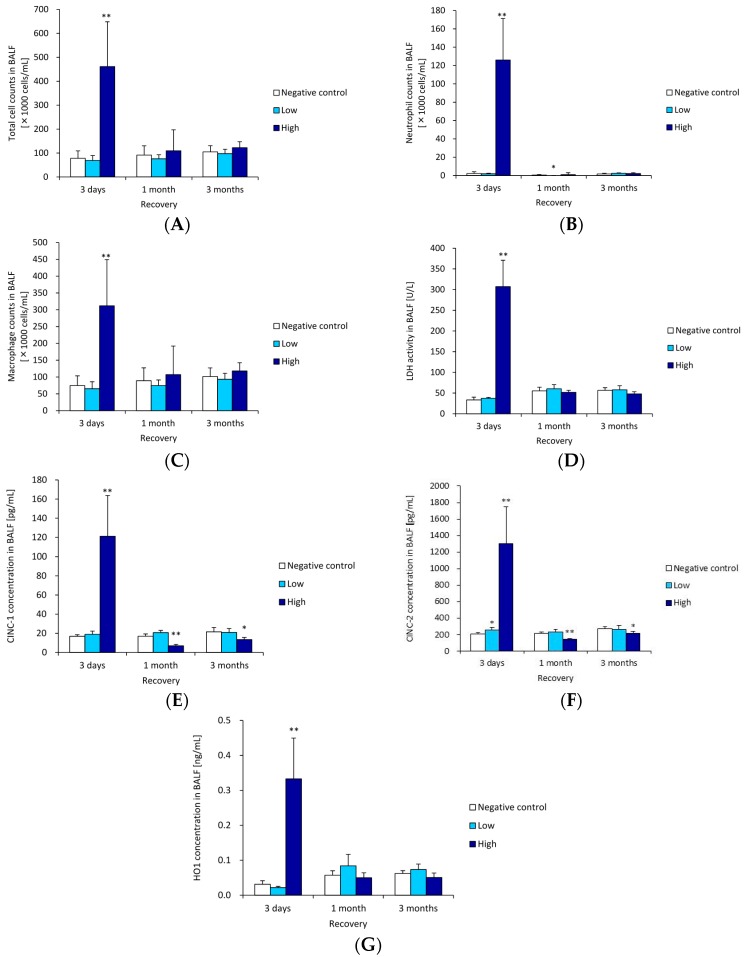
Cell number and cytokine level in BALF following intratracheal instillation of ZnO nanoparticles. (**A**) Total cell count in BALF; (**B**) neutrophil count in BALF; (**C**) macrophage count of in BALF; (**D**) LDH activity in BALF; (**E**) concentration of CINC-1 in BALF; (**F**) concentration of CINC-2 in BALF; (**G**) concentration of HO-1 in BALF. Inhaled ZnO nanoparticles at high concentration transiently induced the influx of inflammatory cells such as neutrophils and expression of CINC-1, CINC-2, and HO-1 in BALF. * indicates *p* < 0.05 compared to negative control. ** indicates *p* < 0.01 compared to negative control.

Figure 4A–C shows the cellular analysis of the BALF following the inhalation of ZnO nanoparticles. The total cell, neutrophil and macrophage counts were higher in the 1 mg groups than in the negative control groups at three days. The value decreased to nearly the negative control level at 1–3 months. The pattern of LDH activity (Figure 4D) was the same as in the cellular analysis. There was a significant increase in LDH activity in the high concentration group at three days, but no significant increase in LDH activity was observed in the ZnO compared to the negative control groups in the other time courses.

### 3.2. CINC Concentration in BALF

Figure 4E,F show the concentrations of CINC-1 and CINC-2 in the BALF following the inhalation of ZnO nanoparticles. Both values in the high concentration groups were significantly elevated at three days post exposure, but the values in the ZnO groups were lower than that in negative control group after one month.

### 3.3. HO-1 Concentration in BALF

Figure 4G shows the concentration of HO-1 in the BALF following the inhalation of ZnO nanoparticles. The concentration of HO-1 in the high concentration groups was higher than that in the negative control group at three days post exposure. There were no significant differences in the concentration of HO-1 between the ZnO and the negative control groups in any other time course.

### 3.4. Histopathological Changes in the Lungs

**Figure 5:**
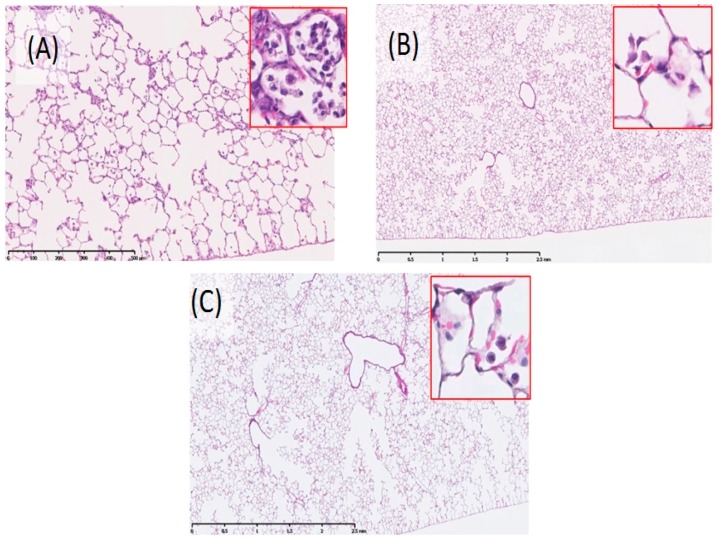
Histological changes in lungs of high dose-inhalation group (40×, inset 200×). (**A**) three days post exposure; (**B**) one month post exposure; and (**C**) three months post exposure. Inflammation of three days after inhalation exposure is milder than that of three days after instillation exposure.

Mild inflammation was induced in small areas of the lungs in the high dose inhalation mice after three days of inhalation (Figure 5A) (Table 2), but there was no significant inflammation after one or three months (Figure 5B,C) (Table 2), nor in any period in the low dose groups. Foamy macrophages and particle-laden macrophages were observed in the alveoli, and some macrophages fused and formed multinucleated cells.

### 3.5. Morphological Features of Alveolar Macrophages by TEM

**Figure 6:**
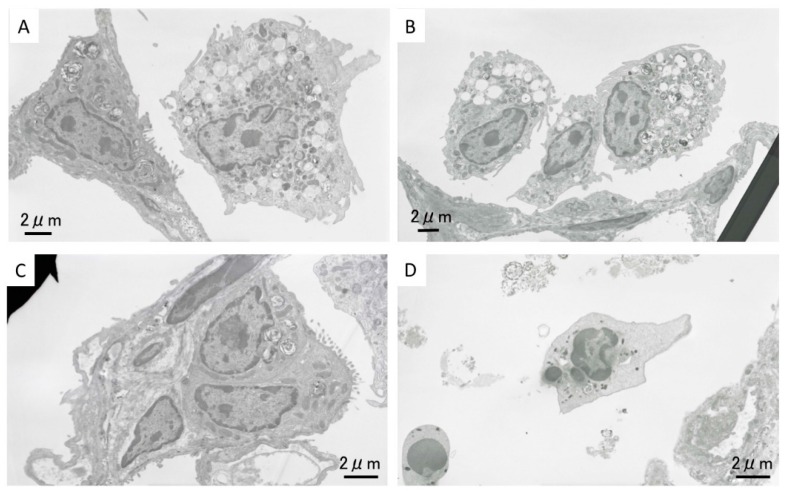
Lung tissue TEM images in the high concentration group at three days following inhalation. (**A**,**B**) Alveolar macrophages with vacuoles in alveolar space; (**C**) Accumulation of alveolar macrophages; (**D**) Neutrophil cells in alveolar space.

TEM images of alveolar macrophages in the high dose ZnO inhalation group lung tissue at three days after exposure are shown in Figure 6A,B. Many vacuoles were observed in the alveolar macrophages. No ZnO nanoparticles were seen in the alveolar macrophages, the same as in the TEM observation in the intratracheal instillation study. Accumulation of alveolar macrophages (Figure 6C) and neutrophil cells (Figure 6D) was observed in the alveolar space.

## 4. Discussion

In the present study, exposure to ZnO nanoparticles following intratracheal instillation and inhalation transiently induced neutrophil inflammation in the rat lung in the acute phase. Many in vivo studies [4,5,6] have shown pulmonary inflammation in animal models. Ho et al. [5] reported that inhalation of not only nanoscale, but also submicron, ZnO induced acute inflammation in the rat lung, and showed that both mass and surface area were affected by the influx of neutrophils in the lung. In vitro studies [1,2,3] have also shown that ZnO induced high cytotoxicity. Lu et al. reported [3] that, among PM and metal oxide nanoparticles, the highest lactate dehydrogenase level was caused by nano-ZnO particles in the A549 cell line (human alveolar adenocarcinoma cell line).

The acute inflammatory level in the present study was approximately two times higher than that by nickel oxide and cerium oxide nanoparticles in our previous studies. We speculate that Zn ions, dissolved by ZnO nanoparticles, affected these high inflammatory responses. Kondura et al. [12] reported that the pulmonary clearance of ZnO nanoparticles in the lung following intratracheal instillation was biphasic, and that both rapid initial and slower terminal half times of ZnO nanoparticles were less than two days. Adamcakova-Dodd [13] showed that 100% of ZnO nanoparticles dissolved within the first 24 h of mixing in an artificial interstitial fluid (pH 4.5). In addition, copper oxide nanoparticles, which are considered to have high solubility, were reported to induce inflammation in the lung through dissolution [14]. These pulmonary responses were based on acute responses, and if the inflammogenic potential of nanoparticles is considered to lead to fibrosis and carcinoma in the lung, sustained inflammation is an important endpoint to speculate the harmful effect of nanoparticles. Even nanoparticles with low toxicity, such as titanium dioxide nanoparticles [15,16,17] and fullerene, induced transient inflammation in rat lung following intratracheal instillation, but not after inhalation. On the other hand, chemicals with high toxicity, such as asbestos and crystalline silica, induced persistent or progressive inflammation mainly by neutrophils, causing irreversible chronic lesions, such as fibrosis and tumors [7,9,18,19].

If the initial lung burden of ZnO following inhalation is calculated by the Multi-Path Particle Model (MPPD model) [20], the initial lung burden in the low and high concentrations following four weeks of inhalation was 0.269 and 1.302 mg/rat (data: low concentration, count median diameter (CMD) 0.126 µm (geometric standard deviation (GSD) 1.77) 2.1 mg/m^3^; high concentration, CMD 0.148 µm (GSD 1.79) 10.4 mg/m^3^), respectively. We think that the initial lung burden in the low and high concentrations approximately correspond to the low and high doses of injected ZnO nanoparticles in the intratracheal instillation study. Compared with either concentrations of ZnO nanoparticles in the inhalation study, inflammatory responses, such as cell analysis, chemokines, and oxidative stress in BALF in both the doses in the intratracheal instillation study were the same, or higher, qualitative level. The bolus effect may have resulted in the values of the data in the intratracheal instillation being higher than those in the inhalation. These tendencies of difference between intratracheal instillation and inhalation studies were also observed in exposure to nickel oxide, titanium dioxide, and multi-wall carbon nanotube (MWCNT) [16,21,22,23,24].

The observation period is important when examining the sustainability of inflammation in an animal model, and we arranged for an observation period of at least three months in the present study. Acute responses were not observed just after the end of exposure to the chemical, and the onset and peak of inflammation were observed after a certain period of observation in animal models. Intratracheal exposure of nickel oxide nanoparticles induced pulmonary inflammation in rats, and the peak of inflammation was at three months post exposure [23]. Sellamuthu et al. [11] reported that the number of neutrophils and the concentration of MCP-1 in the BALF were at the maximum at 16 weeks following inhalation of crystalline silica. Langley et al. [10] performed a six-week inhalation study of silica with 27 weeks of post exposure, and the counts of neutrophils and lymphocytes in the BALF was high at 10 weeks post exposure, although not at four days, and the LDH and protein concentrations in the BALF were significantly higher at 10 and 17 weeks, but not at four days.

Pan et al. [25] performed pulmonary protein profiles in response to ZnO nanoparticles at 24 h and 28 days post exposure following intratracheal instillation, and found that detoxification pathways were activated at the 28-day time-point after exposure, suggesting that insufficient recovery response may develop into irreversible lesions. As we saw no chronic responses through at least three months of observation following both approaches, we assessed that the inflammation induced by ZnO nanoparticles following both approaches was transient. Both pathological features and cell analysis in BALF showed the same transient responses, and both signs were in accordance with each other.

We also examined the concentration of CINC-1 and CINC-2, representative chemokines for neutrophils, in the BALF exposed to ZnO nanoparticles. Exposure to ZnO nanoparticles following intratracheal instillation and inhalation-induced transient elevation of CINC-1 and CINC-2 accompanied by neutrophil influx.

In our previous studies [16,23], the results of neutrophil concentration in BALF showed that the inhalation exposure of NiO and CeO_2_ upregulated the concentration of CINC-1 and CINC-2, but TiO_2_ did not, and the intratracheal instillation of NiO, CeO_2_, and TiO_2_ induced persistent and transient concentration of CINC-1 and CINC-2 in BALF, respectively. These patterns of inflammation by these metal oxide nanoparticles were accompanied by changes in the concentration of CINC-1 and CINC-2. The transient responses in CINC-1 and CINC-2 expression accompanied by neutrophil inflammation in the present study may correspond to previous studies.

HO-1 is known to be one of the representative biomarkers that affect oxidative stress. Li et al. [26] reported that in a dithiothreitol (DTT) assay a quantitative measure of in vitro ROS formation correlated with HO-1 expression in the Abeison murine leukemia virus-induced tumor (RAW264.7) cell line exposed to ultrafine particulate pollutants, in the murine macrophage cell line, human bronchial epithelial cell (BEAS-2B cell) line, and in the human bronchial epithelial cell line.

In the present study, both exposures of ZnO nanoparticles induced transient elevation of HO-1 concentration in BALF. Like the CINC family, the chemicals with high toxicity induced persistent elevation of HO-1 expression, while the chemicals with low toxicity induced transient, or no, elevation. Considering the expression pattern of HO-1, we speculate that ZnO nanoparticles may have a low inflammatory potential.

However, if there is strong oxidative stress in the acute phase of ZnO nanoparticle exposure, there may be a potential for genetic disorders, such as driver gene mutation. As pathological features, only mild and transient hyperplasia was observed, and oxidative DNA injury was not observed (data not shown), suggesting that oxidative stress from ZnO nanoparticles may not be strong, nor would there be induction of genetic disorder. We look forward to future research on the relationship between ZnO nanoparticles and driver gene mutation.

## 5. Methods and Materials

### 5.1. Sample Preparation of ZnO Nanoparticle Suspensions

**Figure 7:**
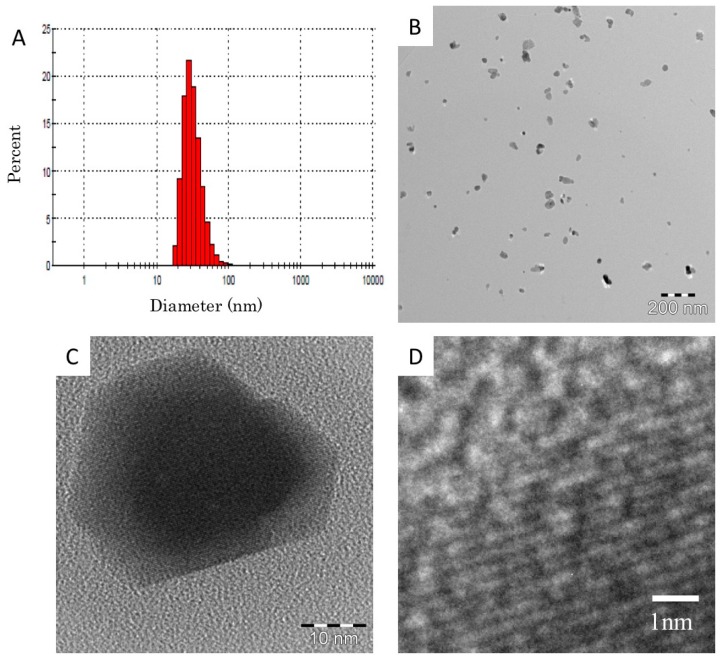
Zinc oxide (ZnO) nanoparticles suspended in distilled water. (**A**) Size distribution of particles was determined by dynamic light scattering technique; (**B**) Low magnification image of ZnO nanoparticles by transmission electron microscopy; (**C**) High magnification TEM image of ZnO nanoparticles; (**D**) Magnified image of (**C**). The crystalline lattice can be clearly observed.

Commercial ZnO nanoparticle dispersion (Sigma-Aldrich Co. LLC., Tokyo, Japan, 51 wt % ZnO) with a water dispersion medium was employed as a source material. The source dispersion contained 2 wt % 3-aminopropyltriethoxysilane as a dispersing agent according to the company’s datasheet. Since no information about the purity was given by the company, we asked Sumika Chemical Analysis Service (Tokyo, Japan) for a purity analysis, and 99.94 wt % purity was reported. The source dispersion was diluted to 10 mg/mL with deionized endotoxin-free water, and was well homogenized by 2 h ultrasonic homogenizing (Branson 5510J-MT, Yamato Scientific Co., Ltd. Tokyo, Japan, 42 kHz 180 W). The prepared dispersion showed a simple secondary particle diameter distribution around the primary particle diameter without agglomeration, as shown in Figure 7A. The average for 500 particles of 35 nm given in Table 3 corresponded to the company’s datasheet (<35 nm). The average secondary particle diameter measured by dynamic light scattering (DLS) for nine samples was 33 nm, which was approximately the same as the primary size, as listed in Table 3, meaning that the ZnO nanoparticles were well dispersed in the suspension.

Though the high concentration dispersion with 10 mg/mL was stable for more than a week, the lower concentration (less than 2 mg/mL) sometimes showed agglomeration (larger than 3 µm diameter) within a week. Therefore, 10 mg/mL dispersions were prepared weekly and diluted to the actual experimental conditions (0.6–5 mg/mL) by 20 min ultrasonic homogenizing just before the experiments.

The ZnO nanoparticle suspensions were observed by a transmission electron microscope (TEM, EM922, Carl Zeiss, Jena, Germany). The accelerating voltage was 160 kV. The TEM specimens were prepared on TEM grids with carbon support films by dropping suspensions and then drying them. TEM images of the ZnO nanoparticle suspensions are shown in Figure 7B. The primary particle size of the ZnO was between 15 and 50 nm. This size distribution was in good agreement with the DLS measurement shown in Figure 7A. Most of the ZnO particles were mono-dispersed, however some made up aggregates from a few primary particles, which were less than 100 nm in size. A high-resolution TEM image of the ZnO primary particles is shown in Figure 7C, and a magnified image of C is shown in Figure 7D. The ZnO particles had a clear crystalline form by the high-resolution TEM image, and it was clarified that no damage was caused by the preparation processes.

### 5.2. Animals

Male Fischer 344 rats (from 9–11 weeks old) were purchased from Charles River Laboratories Japan, Inc. (Yokohama, Kanagawa, Japan). All animals were acclimated in the Laboratory Animal Research Center of the University of Occupational and Environmental Health for at least one week prior to use. All experimental procedures were conducted in accordance with the guidelines described in the Japanese Guide for the Care and Use of Laboratory Animals as approved by the Animal Care and Use Committee, University of Occupational and Environmental Health, Japan (AE12-004, AE12-005).

### 5.3. Intratracheal Instillation of ZnO Nanoparticles

The ZnO nanoparticles were suspended with 0.4 mL distilled water. Rats (12 weeks old) were exposed to 0.2 mg/rat (0.8 mg/kg) or 1 mg/rat (4 mg/kg) of ZnO nanoparticles intratracheally. The negative control groups received distilled water. Low dose (0.2 mg/rat) and high dose (1 mg/rat) were the dose of minimum level which nanomaterials with high toxicity and low toxicity induced minimum persistent inflammation in rat lung following intratracheal instillation [8,27]. Animals were dissected at three days, one week, one month, three months, and six months after the instillation.

### 5.4. Inhalation of ZnO Nanoparticles

ZnO aerosol particles were supplied for the inhalation test at two target concentrations (10 and 2 mg/m^3^). The ZnO nanoparticle suspensions were sprayed with a pressurized nebulizer and dried to disperse the particles in the air flow. They were then delivered into a whole body exposure chamber attached to the rat cages. The setup used here has been described in more detail in our previous papers [28,29]. ZnO suspensions at concentrations of 3–5 and 0.6–0.8 mg/mL were used for the high- and low-dose chambers, respectively. Each of the suspensions was sprayed with the nebulizer at a rate of 0.8 mL/min, using the flow of compressed air at 40 L/min. The droplets generated from the spraying were mixed with 15 L/min of air containing bipolar airborne ions to reduce the electrical charge and, thus the electrically-enhanced loss of droplets. The droplets were successively passed through a heated (150 °C) tube to remove water from them. Clean air was added to the resulting aerosol flow to set the total airflow rate to 100 L/min. This aerosol flow was admitted into the exposure chamber for 6 h per day. The inhalation test period was four weeks.

A particle size spectrometer (model 1000XP WPS, MSP Corp., Shoreview, MN, USA) was used to measure the aerosol particle size distribution in the exposure chambers twice an hour. A small amount of the aerosol was sampled periodically outside of the chamber. The particles in the aerosol were deposited onto a Cu TEM grid and subjected to TEM observation. Particles were also collected on a fibrous filter and weighed to determine the mass concentration of the aerosols in the chambers, which took place 3–5 times per day. After the inhalation test period, the rats were dissected after three days, one month, and three months of recovery.

**Figure 8:**
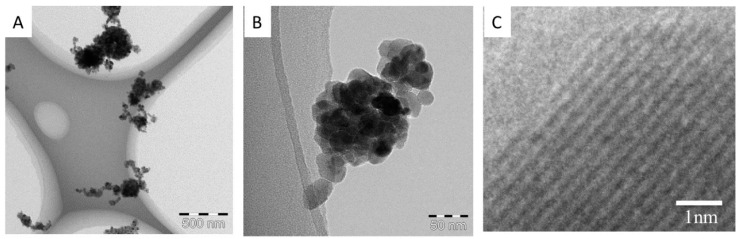
Inhaled ZnO nanoparticles in exposure chambers by transmission electron microscopy (**A**,**B**); (**C**) High magnification TEM image of ZnO nanoparticles.

The particle size distributions of the ZnO aerosols in both the high- and low-dose chambers were sufficiently stable for 6 h on every day of the test period The geometric mean diameter of the aerosol particles averaged for the test period was 148 ± 14 nm (*n* = 480) for the high-dose chamber, and 126 ± 11 nm (*n* = 480) for the low-dose chamber. The average mass concentrations in the test period were 10.4 ± 1.39 mg/m^3^ (*n* = 73) and 2.11 ± 0.45 mg/m^3^ (*n* = 70) for the high- and low-dose chambers, respectively. Figure 8A–C show typical TEM images of the ZnO aerosol particles sampled from the high dose chamber. The particles were aggregates, and their sizes were mostly in the range of 50 and 300 nm, with a peak at around 150 nm. This was consistent with the result obtained with the particle size spectrometer. Crystal lattices can clearly be seen in a high resolution TEM image (Figure 8C), indicating that the aerosol generation process did not cause any damage to the ZnO particles.

### 5.5. Animals after the Inhalation and Intratracheal Instillation Studies

There were 10 rats, classified into two subgroups of five animals each, in the negative control, low-dose, and high-dose groups in each time course for BALF and lung tissue analysis. In the first subgroups (five animals in each dose group), the lungs were divided into right and left lungs. Histopathological evaluation was performed with the left lung inflated and fixed by 10% formaldehyde. In the second subgroups (five animals in each dose group), the lungs were inflated with physiological saline with 20 mL water under a pressure of 20 cm, and recovered fluid was collected from whole lung divided two to three times. Between 15 and 18 mL of the recovered fluid was collected in collection tubes by free fall. Analysis of cytokine was performed with BALF.

### 5.6. Analysis of Inflammatory Cells in BALF

From 10–13 mL of recovered BALF was centrifuged at 400× *g* at 4 °C for 15 min. The supernatant was transferred to a new tube and used for measuring the cytokines in the BALF. The pellets were washed by suspension with polymorphonuclear leukocyte (PMN) Buffer (137.9 mM NaCl, 2.7 mM KCl, 8.2 mM Na_2_HPO_4_, 1.5 mM KH_2_PO_4_, 5.6 mM C_6_H_12_O_6_) and centrifuged at 400× *g* at 4 °C for 15 min. After the supernatant was removed, the pellets were resuspended with 1 mL of PMN Buffer. The total cell numbers in the BALF was counted by Celltac (Nihon Kohden, Tokyo, Japan), and the cells were splashed on a slide glass using cytospin. After the cells were fixed and stained with Diff-Quik (System Corporation, Hyogo, Japan), the number of neutrophils and alveolar macrophages was counted by microscopic observation.

### 5.7. Chemokines, LDH, and HO-1 in BALF

The concentrations of rat chemoattractant (CINC)-1 and rat CINC-2α/β in the BALF supernatant were measured by ELISA kits #RCN100 and #RCN200 (R and D Systems, Minneapolis, MN, USA), respectively. The concentrations of rat HO-1 in BALF supernatant were measured by an ELISA kit, ADI-EKS-810A (Enzo Life Sciences, Farmingdale, NY, USA), and the activity of released LDH in BALF supernatant was measured by a Cytotoxicity Detection Kit^PLUS^(LDH) (Roche Diagnostics GmbH, Mannheim, Germany). The LDH activity in BALF supernatant was determined in an enzymatic test. All procedures were performed according to the manufacturer’s instructions.

### 5.8. Histopathology

The lung tissue, which was inflated and fixed with 10% formaldehyde under a pressure of 25 cm water, was dehydrated and embedded in paraffin, and 5 µm-thick sections were cut from the lobe, then stained with hematoxylin and eosin.

## 6. TEM Experimental Methods

Lung tissues were observed by TEM after the inhalation and intratracheal instillation studies. The TEM specimen preparation method is described below. The lung tissues were fixed by a perfusion system of a 4% paraformaldehyde solution, and then were post-fixed in a 1% osmium tetroxide solution. They were dehydrated in ethanol subsequently, followed by embedding in epoxy resin. Ultrathin sections were cut by using a diamond knife using microtomy. The specimens were stained with a 2% uranyl acetate solution, and then a mixed solution of 0.3% lead nitrate and 0.3% lead acetate. All of them were prepared at room temperature. Conventional TEM observation was performed with an H-7600 (Hitachi High-Technologies Corp., Tokyo, Japan). The accelerating voltage was 80 kV.

### Statistical Analysis

Analysis of Mann-Whitney test were applied where appropriate to determine individual differences using a computer statistical package (SPSS, SPSS Inc., Chicago, IL, USA).

## 7. Conclusions

We conducted inhalation and intratracheal instillation of ZnO nanoparticles in order to examine their toxicity. In the intratracheal instillation study, F344 rats were exposed to 0.2 or 1 mg of ZnO nanoparticles. In the inhalation study, rats inhaled ZnO nanoparticles at a maximum concentration of 10 mg/m^3^ for four weeks. The intratracheal instillation and the inhalation of a high dose of ZnO nanoparticles caused a transient increase in neutrophil influx in the lung and a transient increase in concentration of CINC-1, CINC-2, and HO-1 in BALF in the acute phase. These parameters returned to control level in the chronic phase, and reversible inflammation of neutrophils in the lung was observed by both approaches. The transient inflammation in the lung exposed to ZnO nanoparticles suggests that ZnO nanoparticles may have a low toxic potential.

## Figures and Tables

**Table 1:** Pathological features in the rat lung following intratracheal instillation of ZnO nanoparticles.

Time	3 Days (*n* = 5)			1 Week (*n* = 5)			1 Month (*n* = 5)			3 Months (*n* = 5)			6 Months (*n* = 5)		
Pathological Feature	Negative Control	ZnO 0.2 mg	ZnO 1.0 mg	Negative Control	ZnO 0.2 mg	ZnO 1.0 mg	Negative Control	ZnO 0.2 mg	ZnO 1.0 mg	Negative Control	ZnO 0.2 mg	ZnO 1.0 mg	Negative Control	ZnO 0.2 mg	ZnO 1.0 mg
Macrophage infiltration in alveolar space	−	++	++	−	+	+	−	±	±	−	− ~ ±	− ~ ±	−	− ~ ±	− ~ ±
Inflammatory cell infiltration in alveolar space	−	++	+++	−	+	+	−	−	− ~ ±	−	−	−	−	−	−
Infiltration in interstitial area	−	+	++	−	±	±	−	−	− ~ ±	−	−	−	−	−	−
Hyperplasia of bronchiolar epithelial cell	−	+	+	−	− ~ ±	− ~ ±	−	− ~ ±	− ~ ±	−	−	− ~ ±	−	−	− ~ ±
Hyperplasia of alveolar epithelial cell	−	++	++ ~ +++	−	±	±	−	−	−	−	−	−	−	−	−
Fibrosis	−	± ~ +	± ~ +	−	±	±	−	−	− ~ ±	−	−	− ~ ±	−	−	− ~ ±
Tumor	−	−	−	−	−	−	−	−	−	−	−	−	−	−	−

Grade of changes: −, none; ±, minimum; +, mild; ++, moderate; +++, remarked.

**Table 2:** Pathological features in the rat lung following inhalation of ZnO nanoparticles.

Time	3 Days (*n* = 5)			1 Month (*n* = 5)			3 Months (*n* = 5)		
Pathological Feature	Negative Control	ZnO Low	ZnO High	Negative Control	ZnO Low	ZnO High	Negative Control	ZnO Low	ZnO High
Macrophage infiltration in alveolar space	−	+	++	−	±	+	−	±	±
Inflammatory cell infiltration in alveolar space	−	−	− ~ ±	−	−	−	−	−	−
Infiltration in interstitial area	−	−	− ~ ±	−	−	−	−	−	−
Hyperplasia of bronchiolar epithelial cell	−	−	− ~ ±	−	−	−	−	−	−
Hyperplasia of alveolar epithelial cell	−	−	−	−	−	−	−	−	−
Fibrosis	−	−	−	−	−	−	−	−	−
tumor	−	−	−	−	−	−	−	−	−

Grade of changes: −, none; ±, minimum; +, mild; ++, moderate.

**Table 3:** Physicochemical properties of zinc oxide (ZnO) nanoparticles used in the experiment.

Nanomaterials	ZnO Nanoparticle
Manufacturer	Sigma-Aldrich Co. LLC.
Chemical formula	ZnO
Primary diameter	35 nm
Specific surface area	31 m^2^/g
Shape	Polyhedral roughly round
Secondary diameter (DLS)	33 nm
Purity	99.94 wt %
Bulk density	5.6 g/cm^3^
Solubility	high
